# CMOS Implementation of ANNs Based on Analog Optimization of N-Dimensional Objective Functions

**DOI:** 10.3390/s21217071

**Published:** 2021-10-25

**Authors:** Alejandro Medina-Santiago, Carlos Arturo Hernández-Gracidas, Luis Alberto Morales-Rosales, Ignacio Algredo-Badillo, Monica Amador García, Jorge Antonio Orozco Torres

**Affiliations:** 1 Department of Computer Science, CONACYT-INAOE (Instituto Nacional de Astrofísica, Óptica y Electrónica), Santa María Tonanzintla, Puebla 72840, Mexico; algredobadillo@inaoep.mx; 2 Physical-Mathematical Science Department, CONACYT-BUAP, Puebla 72570, Mexico; 3 Faculty of Civil Engineering, CONACYT-Universidad Michoacana de San Nicolás de Hidalgo, Morelia 58000, Mexico; lamorales@conacyt.mx; 4 Instituto Tecnológico Superior de Rioverde, Tecnológico Nacional de México, San Luis Potosi 79610, Mexico; mony_951@hotmail.com; 5 Campus Tuxtla Gutiérrez, Tecnológico Nacional de México, Tuxtla Gutiérrez 29050, Mexico; jorge.ot@tuxtla.tecnm.mx

**Keywords:** CMOS circuit, analog system, signal processing, learning algorithm, artificial neural network

## Abstract

The design of neural network architectures is carried out using methods that optimize a particular objective function, in which a point that minimizes the function is sought. In reported works, they only focused on software simulations or commercial complementary metal-oxide-semiconductor (CMOS), neither of which guarantees the quality of the solution. In this work, we designed a hardware architecture using individual neurons as building blocks based on the optimization of n-dimensional objective functions, such as obtaining the bias and synaptic weight parameters of an artificial neural network (ANN) model using the gradient descent method. The ANN-based architecture has a 5-3-1 configuration and is implemented on a 1.2 μm technology integrated circuit, with a total power consumption of 46.08 mW, using nine neurons and 36 CMOS operational amplifiers (op-amps). We show the results obtained from the application of integrated circuits for ANNs simulated in PSpice applied to the classification of digital data, demonstrating that the optimization method successfully obtains the synaptic weights and bias values generated by the learning algorithm (Steepest-Descent), for the design of the neural architecture.

## 1. Introduction

The design process of complementary metal-oxide-semiconductor (CMOS) circuits consists of defining circuit inputs and outputs, hand calculations, circuit simulations, circuit layout, simulations including parasitics, reevaluation of circuit inputs and outputs, fabrication, and testing [1]. Circuit specifications are rarely defined; they can change as the design of the circuit or application progresses. This is the result of seeking the reduction of costs and improving performance of the design in its manufacture; it can also be due to the chip type or needs of the end-user. In most cases, it is not possible to make major changes to the design once the chip is in production (www.mosis.org, accessed on 18 October 2021). The characteristics of the CMOS allow the integration of logic functions with high density in integrated circuits. Due to this, CMOS has become the most widely used technology within very large-scale integration (VLSI) chips [2,3].

CMOS is used in static random access memory (RAM), digital logic circuits, microprocessors, microcontrollers, image sensors, and the conversion of computer data from one file format to another. Most configuration information on newer central processing units (CPUs) is stored on one CMOS chip. The configuration information on a CMOS chip is called the real-time clock/nonvolatile RAM (RTC/NVRAM) chip, which works to retain data when the computer is shut off.

CMOS transistors are very famous because they use electrical power efficiently. They use no electrical supply whenever they are alternating from one condition to another. Furthermore, the complementary semiconductors work mutually to stop the o/p voltage. The result is a low-power dissipation VLSI design; for this reason, these transistors have changed other earlier designs like charge-coupled devices (CCDs) within camera sensors, which are used in most of the current processors. CMOS memories within a computer are a kind of non-volatile RAM that store BIOS settings, as well as time and date information.

Although most of the advances in neural networks have resulted from theoretical analysis or computer simulations, many of the potential advantages of artificial neural networks (ANNs) are expected to be implemented in hardware. Fortunately, the rapid advance in VLSI technology has made many of the previously impossible ideas now feasible to realize. Therefore, in this work, the numerical analysis of an n-dimensional objective-function optimization strategy is presented. This strategy, based on gradient descent, and its software implementation for the optimization of the weight and bias parameter values in multilayer ANNs, with the purpose of achieving their convergence, are presented as well [1,4,5]. A hardware implementation using CMOS transistors with operational amplifiers as its base elements is designed from the resulting ANN training model. A case study for the classification of digital patterns using multilayer ANNs is presented, which illustrates the implementation of the whole process. The electronic simulation of the developed integrated circuit in PSpice shows it is feasible to consider that this optimization method is efficient. In summary, the contributions of this work are as follows, sorted by relevance:

1. The design of circuits, using CMOS transistors through operational amplifiers as base elements, of the training model obtained from the parameter optimization, applied to multilayer ANNs.
2. An analysis of the designed integrated circuit (ANN hardware) based on the electronic simulation results in Pspice.
3. The software simulation of the weight and bias parameter optimization, with the purpose of achieving the convergence of a multilayer ANN (the automatic learning algorithm) based on gradient descent.
4. A multilayer ANN hardware case study simulation, designed to classify digital patterns to identify whether data is correctly sent.

Optimization techniques are ubiquitous in the field of ANNs. Generally, most learning algorithms used for training ANNs can be formulated as optimization problems because they are based on the minimization of an error function (Lyapunov function), called E(x), where $x={\left[x_{1},x_{2},\ldots ,x_{n}\right]}^{T}$ is the parameter vector. One of the most important fundamentals required in the learning algorithms is the calculation of the gradient of a specific objective function ∇E(x) with respect to these parameters (synaptic weights $w_{ij}$, gain parameter $\gamma_{i}$, decay constant characteristic $\alpha_{i}$, and Lagrange multipliers) [6,7,8].

It is well known that almost all optimization problems are solved numerically by iterative methods. Some of these iterative methods can be considered as a discrete-time realization of continuous-time dynamic systems. Specifically, a continuous-time (analog) dynamic system is described by a set of ordinary differential Equations (usually nonlinear), while a discrete-time dynamic system involves systems of differential equations. The advantages of using a system of differential equations are:

1. Real-world problems can be modeled with the use of differential equations, which allow humans to understand various fundamental laws of science [7]. Therefore, the simulation or implementation of a system of differential equations allows real-time optimization problems to be solved; this is due to the extraordinary parallel operation of the calculation units and the convergence properties of the neural systems.
2. The convergence properties of a continuous-time system are better because the learning rates can be set to be arbitrarily large without affecting the system stability. In contrast, in a discrete-time system, we must bound the control parameters in a small interval or, otherwise, the system may become unstable (i.e., the algorithm diverges).
3. A dynamic system implemented with basic differential equations exhibits more robustness to certain parameter variation [9,10].
4. Sometimes, continuous-time systems link different discrete-time iterative systems, as special cases for discretization, leading to the development of new iterative algorithms. Consequently, the simulation of continuous-time dynamic systems is more sophisticated and has faster techniques.

This work is organized as follows. Section 2 of this article shows the mathematical foundations of the Steepest-Descent objective function optimization method. Section 3 shows the analysis and development of the proposed model for the implemented circuit. Section 4 exemplify the design of integrated circuits for ANN architectures with CMOS transistors. Section 5 shows the application of the ANN to digital pattern recognition. Section 6 presents a comparison between ours and other related works. Finally, Section 7 expresses the conclusions of the work.

## 2. Mathematical Foundations

The basic principles of optimization were discovered in the 17th century by scientists and mathematicians such as Kepler, Fermat, Newton, and Leibniz [11,12]. Since 1950, these principles have been rediscovered for their implementation in digital computers. The progress of this effort significantly stimulated the search for new algorithms, and the field of optimization theory came to be recognized as one of the best fields of mathematics. Currently, researchers using ANNs have access to a wide range of theories that can be applied to the training of these networks.

A well-known mathematical algorithm used for solving the optimization problems is described below: the basic iterative Steepest-Descent algorithm [13,14]. The top-down method, also known as the gradient method, is defined in an n-dimensional space and is one of the oldest techniques used for minimizing a given cost function. This method forms the basis of the direct methods used in the optimization of restricted and unrestricted problems. Furthermore, it is the most used technique for non-linear optimization. In other words, what is sought is a value of *x* that minimizes the cost function E(x).

Let us consider the following unconstrained optimization problem: find a vector $x\in R^{n}$ that minimizes the real-valued scalar function

(1) E=E(x),

This function is called the cost, objective, or energy function and *x* is an n-dimensional vector called the design vector. Minimizing a function is the same as maximizing the negative of the function, so there is no loss of generality in our considerations.

The point $x^{*}$ is a global minimizer for E(x) if $E\left(x^{*}\right)\leq E\left(x\right)$ for all $x\in R^{n}$, and a strict local minimizer if the relation $E\left(x^{*}\right)\leq E\left(x\right)$ holds for a ball $B(x^{*};\epsilon )$.

Assuming that the first and second derivates of E(x) exist, a point $x^{*}$ is a strict local minimizer of E(x) if the gradient is zero (i.e., $\nabla E(x^{*})=0$) and the Hessian matrix is positive definite (i.e., $x^{T}\nabla^{2}E\left(x^{*}\right)x>0$).

The above statement can be formulated as a theorem on necessary and sufficient conditions for a strict local minimizer: Let $\nabla^{2}E\left(x\right)$ be nonsingular for point $x^{*}$. Then, we have $E(x^{*})$ < E(x) for every *x* in $0<||x-x^{*}\left|\right|<\epsilon$ with some ϵ>0, if $\nabla E(x^{*})=0$ and $\nabla^{2}E\left(x^{*}\right)$ is symmetric and positive definite.

Considering an optimization problem, which has a cost function:

(2) $$E\left(x\right)\;\mathrm{subject}\;\mathrm{to}\;x\in R^{n},$$

Suppose there exists, at the same time, a gradient vector and the Hessian matrix of the objective function (i.e., they can be evaluated analytically), then this involves generating a sequence of search points $x^{\left(k\right)}$ through the iterative procedure:

(3) $$x^{\left(k+1\right)}=x^{\left(k\right)}+\eta^{\left(k\right)}*d_{k},\;x\left(0\right)=x^{\left(0\right)},\;\left(k=0,1,2,\ldots \right),$$

where $\eta^{\left(k\right)}>0$ determines the length of the step (learning rate) to be taken in the direction of the vector $d_{k}≅\Delta x^{\left(k\right)}$ (search direction). In numerical optimization, there are different techniques to calculate the parameter $\eta^{\left(k\right)}$ and the direction of the vector $d_{k}$. For convenience, four basic methods are presented:

1. Gradient method (Steepest-Descent) [6,12,15], where the direction is defined as:
  (4) $$d_{k}:=-\nabla E\left(x^{\left(k\right)}\right),$$
2. Newton’s method [11], in which the search direction is determined by:
  (5) $$d_{k}:=-{\left[\nabla^{2}E\left(x^{\left(k\right)}\right)\right]}^{-1}\nabla E\left(x^{\left(k\right)}\right),$$
3. Since the calculation of the Hessian inverse matrix ${\left[\nabla^{2}*E\left(x^{\left(k\right)}\right)\right]}^{-1}$ can be slightly complicated, a symmetric positive matrix of dimension n×n is defined, which is called $H_{k}$.
  (6) $$H_{k}≅{\left[\nabla^{2}E\left(x^{\left(k\right)}\right)\right]}^{-1},$$
  Applying the quasi-Newton method [11,12,15] the search direction is determined by:
  (7) $$d_{k}=-H_{k}\nabla E\left(x^{\left(k\right)}\right),$$
4. The conjugate gradient method [11,12,15,16,17] calculates the current search direction $d_{k}$ as a linear combination of the current gradient vector and the previous search direction. This simple way to find the direction is calculated by:
  (8) $$d_{k}=-\nabla E\left(x^{\left(k\right)}\right)+\beta_{k}d_{k+1},\;\left(k=0,1,2,\ldots \right),$$
  with $d_{0}=-\nabla E\left(x^{\left(0\right)}\right)$, where $\beta_{k}$ is a scalar parameter that ensures that the vector sequence $d_{k}$ satisfies the condition of mutual conjugation.

The length of the step $\eta^{(}k)$ is usually determined by these methods using one of the following techniques: (1) Minimization along the line, and (2) A fixed step size in one dimension. In the next subsection, the mathematical development of the continuous-time interactive algorithm for calculating the local minimum of a cost function is described.

### 2.1. Continuous-Time Iterative Algorithm

The gradient descent method discussed above can be written as:

(9) $$x^{\left(k+1\right)}:=x^{\left(k\right)}-M_{k}\nabla E\left(x^{k}\right),$$

where $M_{k}$ is symmetrically defined as a positive matrix of dimension n×n. The appropriate choice of matrix $M_{k}$ is critical, based on the convergence properties of the algorithm. The discrete-time minimization algorithm determines the local minimum of the cost function E(x) as the limit of the sequence $E\left(x^{\left(k\right)}\right)\left(k=0,1,2,\ldots \right)$ where $x^{\left(0\right)}$ is an initial estimate of the local minimizer $X^{*}$.

These iterative algorithms generate a sequence of points $\left\{x^{\left(k\right)}\right\}$ and a search direction $d_{k}≅\Delta x^{\left(k\right)}$ through a discrete-time approximation for some continuous-time trajectory from the starting point $x^{\left(0\right)}$ to the minimum point $x^{*}$ (stationary). The continuous-time trajectory x(t) is usually determined by a system of differential equations as follows:

(10) $$\frac{dx_{j}}{dt}=-\sum_{i=1}^{n}\mu_{ji}\frac{\partial E(x)}{\partial x_{i}},\;X_{j}\left(0\right)=x_{j}^{\left(0\right)},\;\left(j=1,2,\ldots ,n\right),$$

This expression can be written more compactly in the form of a matrix:

(11) $$\frac{dx}{dt}=-\mu \nabla E\left(x\right),\;x\left(0\right)=x^{\left(0\right)},$$

where $x\left(t\right)\in R^{n}$, μ(x,t) is a positive definite matrix of dimension n×n whose inputs are generally dependent on time, and the variable $x\left(t\right)={\left[x_{1}\left(t\right),x_{2}\left(t\right),\ldots ,x_{n}\left(t\right)\right]}^{T}$. Determining the positive matrix μ requires system stability. The next subsection describes a basic gradient system used to determine the direction of gradient change of a system of differential equations.

### 2.2. Basic Gradient System

Considering the simple case for which the matrix μ(x,t) is reduced to a positive scalar function μ(t), the system of differential equations according to (10) is simplified to:

(12) $$\frac{dx_{j}}{dt}=-\mu \left(t\right)\frac{\partial E}{\partial x_{j}},$$

with $x_{j}\left(0\right)={x_{j}}^{\left(0\right)}\;\left(j=1,2,\ldots ,n\right)$; μ(t)>0∈C is a constant and theoretically it can be a large arbitrary set; the learning relationship $\eta^{\left(k\right)}$, in a discrete-time slope algorithm (ascending slope), is limited to a small interval to ensure that the algorithm converges. The previous system of differential equations is called the basic dynamic gradient system. Continuous-time gradient downward methods employ (12), since the search of −∇E(x) is in the direction of maximum negative change of the objective function E(x) at some point.

An interesting aspect of the method is the fact that the direction determined by the discrete-time iterative algorithm is slightly oscillatory. In contrast, the direction obtained by the continuous-time gradient method is monotonous. In other words, the oscillation effects can be eliminated using a continuous-time gradient system. As described, the main objective of this algorithm is to find a value of *x* that minimizes E(x).

## 3. Analysis and Development

**Figure 1:**
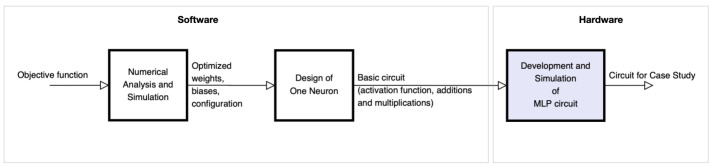
Block diagram of the design and development of the proposed methodology.

From the optimized model of the ANN, we carried out the CMOS circuit design based on the operational amplifier to design the base cell called perceptron, which is commonly used in multi-layer perceptron (MLP) architectures. Figure 1 shows the block diagram of the proposed methodology for the development of the circuit. Block 1 represents the numerical analysis stage and the simulation of the optimization of the objective function (obtaining synaptic weights and bias) for the ANN, based on gradient. Block 2 is focused on the design of the base neuron (perceptron) at the circuit level. Finally, block 3 shows the development and simulation of the complete MLP circuit to obtain the neural network behavior proposed in the case study.

All the optimization algorithms described above employ a system of first-order differential equations. Now, we need to apply the optimization to a system of second-order differential equations. Therefore, to improve the convergence properties, we can use a system of higher-order ordinary differential equations by considering the system of second-order differential Equations (1), (3) and (8) as follows:

(13) $$\delta \left(t\right)\frac{d^{2}x}{dt^{2}}=-\gamma \left(t\right)T\frac{dx}{dt}-\nabla E\left(x\right),$$

with initial conditions $x\left(0\right)=x^{\left(0\right)}$, $\left(\frac{dx}{dt}\right)\left(0\right)=x^{\prime }\left(0\right)=x^{\prime (0)}$, where $x\left(t\right)\in R^{n}$, δ(t) and γ(t) are positive functions with real values for t≥0, and $T=\left[T_{ij}\right]$ is a positive definite symmetric matrix of dimension n×n.

A simple case is when δ, γ, and *T* are constant; for example: $\delta \left(t\right)=\delta_{0}\geq 0$, $\gamma \left(t\right)=\gamma_{0}>0$, $T=\left[T_{ij}\right],\;\mathrm{and}\;T_{ij}$ is constant for i,j=1,2,…,n. However, in general, the matrix T=T(x,t) depends on time. A particular choice of the parameters of (13) makes it possible to obtain almost all first-order methods. For example, when δ(t)=0, the conditions are the following:

1. For T=I (identity matrix) and $\gamma \left(t\right)=\mu^{\left(-1\right)}>0$, the gradient descent method is applied.
2. For $T=\nabla^{2}E\left(x\right)$ (Hessian matrix) and γ(t)=1, Newton’s method is applied.
3. For $T=[\nabla^{2}E\left(x\right)+v\left(t\right)I]$ and γ(t)=1, the Levenberg–Marquardt method [11,12,15,16,17] is applied.

The system of second-order equations is inspired by classical mechanics and has the following physical interpretation [6]: (13) represents Newton’s second law (mass × acceleration = force) for a mass particle δ(t) moving in a space $R^{n}$ subject to a force −∇E(x) given by the potential E(x) and with force $-\gamma \left(t\right)T\left(\frac{dx}{dt}\right)$. Since γ(t)>0, the force $-\gamma \left(t\right)T\left(\frac{dx}{dt}\right)$ is dissipative and γ(t) is the coefficient of friction. Generally, the mass coefficient of δ(t) and the coefficient of friction γ(t) are constant in time or tend to zero as time approaches infinity.

The application of a system of second-order differential equations has several important advantages over a system of first-order differential equations, which are:

1. Because of the initial force, the local minimum of the objective function E(x) can be avoided by an appropriate choice of parameters, and the network can find an overall minimum, although this cannot be guaranteed.
2. The second-order differential equations have better flexibility. For example, for the same starting point x(0) different from the selection of $\left(\frac{dx}{dt}\right)\left(0\right)$, they can lead to a different local minimum. That is, we changed coefficients γ and δ making it possible to reach a local minimum from the same initial condition $\left(x\left(0\right),x^{\prime }\left(0\right)\right)$. Thus, in a system of differential equations of the form given by (13), an additional control of the solution is provided.
3. A system of second-order differential equations may have a better property of convergence. Therefore, responses can be obtained in its trajectory.

To evaluate the behavior of the gradient descent algorithm, we performed two simulations. In the first example, we have a single-variable objective function for which the determination of the local minimum depends on the initial condition of the variable. A two-variable objective function (which can fall into a saddle point or a local minimum) is described in the second example. This case can be avoided by an appropriate choice of the initial condition of the variables. The results obtained from the applications of the algorithm and the initial conditions are shown in Table 1 and Table 2, respectively.

Applying (13), the optimization problem (one-dimensional) is:

(14) $$\delta_{0}\frac{d^{2}x}{dt^{2}}=-\gamma_{0}\frac{dx}{dt}-\mu_{0}\left(4*sin\left(x\right)cos\left(x\right)+0.2x\right),$$

Applying (13), the optimization problem (n-dimensional) has the following form:

(15) $$\delta_{0}\left(\frac{d^{2}x_{1}}{dt^{2}}\right)=-\gamma_{0}\left(\frac{dx_{1}}{dt}\right)-\mu_{0}*cos\left(x_{1}\right)*sin\left(x_{2}\right),$$

(16) $$\delta_{0}\left(\frac{d^{2}x_{2}}{dt^{2}}\right)=-\gamma_{0}\left(\frac{dx_{2}}{dt}\right)-\mu_{0}*cos\left(x_{2}\right)*sin\left(x_{1}\right),$$

When a search of the local minimum of a particular objective function of two or more variables is performed, it is necessary to avoid falling into the saddle points caused by the initial conditions of the variables when calculating the solution. In Table 1, the results obtained for examples 1 and 2 show how we can improve the convergence properties of an objective function with one or two variables. In general, an n-dimensional objective function will work, using the proposed system of higher-order ordinary differential equations. In the following section, we will show how this approach can be applied. We will do this through a case study for its application to an ANN implemented using analog systems and CMOS circuits.

## 4. Circuit Design: A Case Study of ANNs Implemented in CMOS Circuits

**Figure 2:**
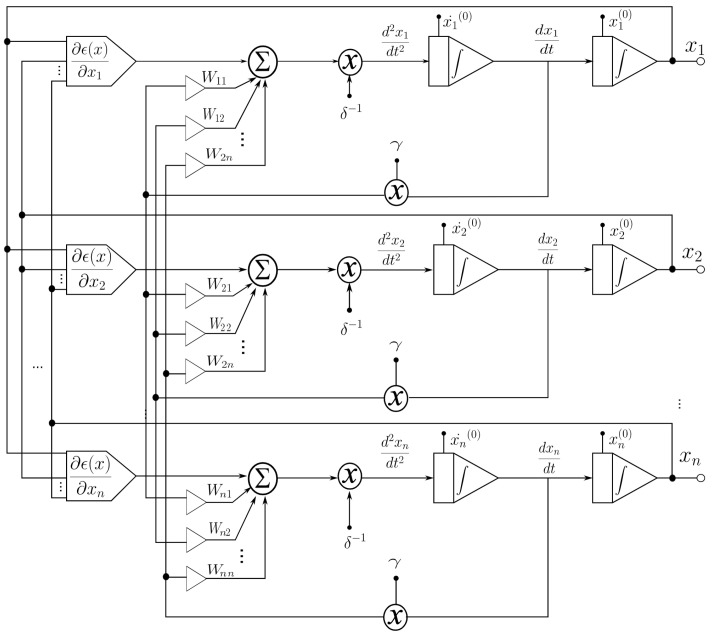
Block diagram for applications to ANNs.

**Figure 3:**
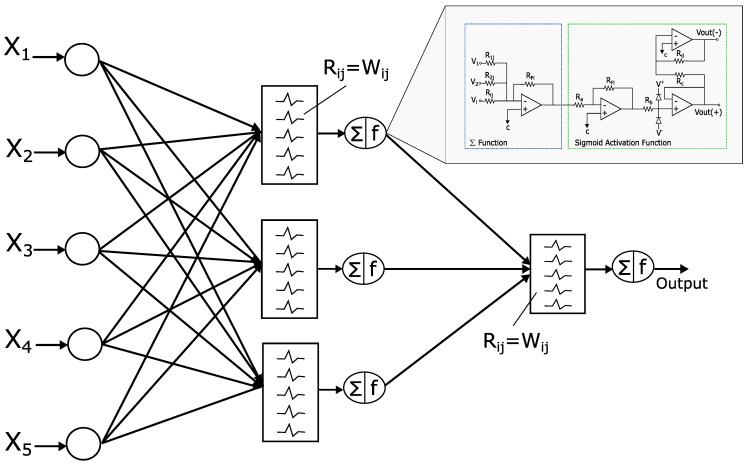
Complete circuit based on ANN.

**Figure 4:**
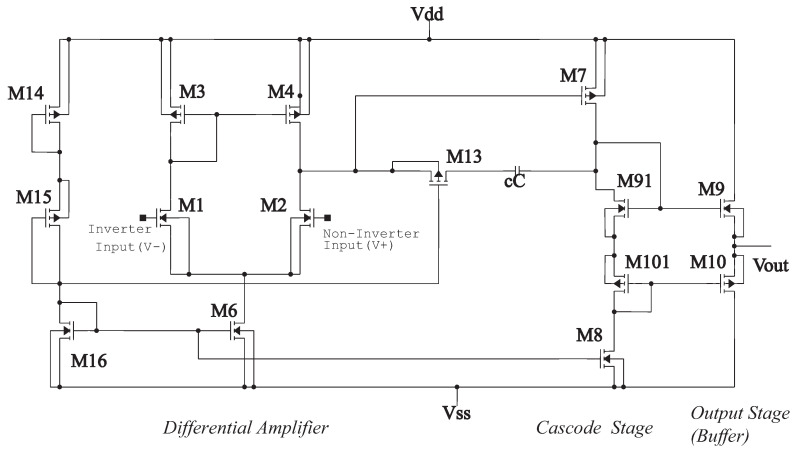
Operational amplifier 1.2 μm.

In this section, the application of the proposed system to an ANN [1,4,5] is described. An analog multiplier multiplies the outputs of a voltage adder concerning a constant. The voltage adder generates the sum of the voltages of the matrix $W_{ij}$, which represents the synaptic weight matrix (Figure 2), and the signal of the non-linear function generator. We used operational amplifiers (op-amps) to design each base neuron that composes the neural architecture. It means that we implemented the structure of the neural circuit of Figure 2 to produce the ANN represented in Figure 3. We remark that each neuron node has two parts: a sum function and an activation function (sigmoid). We implemented the first one with an op-amp inverting adder (designed based on Figure 4) and the second one with an array of op-amps and voltage limiters (diodes).

Figure 2 shows the block diagram for the function, which consists of two continuous-time integrators (whose response depends on the feedback network), one analog multiplier, one summing amplifier, and one non-linear function generator for calculating the gradient of the objective function at the circuit level. The optimized parameters $x\left(t\right)={\left[x_{1}\left(t\right),x_{2}\left(t\right),\ldots ,x_{n}\left(t\right)\right]}^{T}$ are the output signals of the integrator. This circuit is characterized by having a more robust output (insensitive to small perturbations) with respect to the parameter variation. The function generator is the only one that precisely calculates the gradient of the objective function.

**Figure 5:**
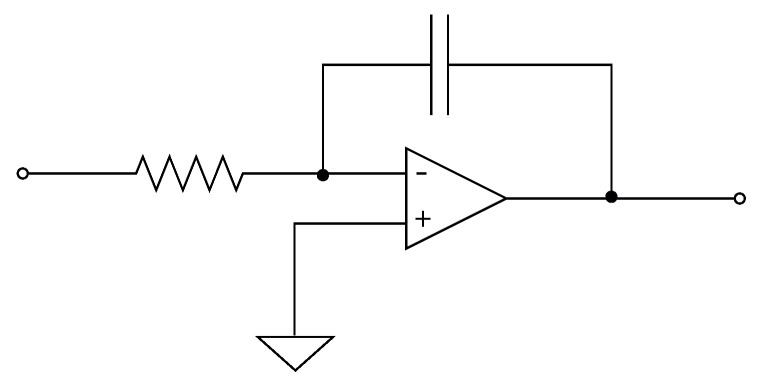
Integrator Circuit.

**Figure 6:**
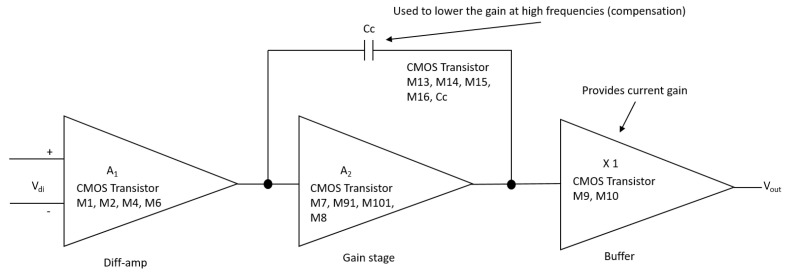
Block diagram of the 1.2 μm operational amplifier.

According to Figure 2, the design of the analog neural network consists of the development of the integrator circuit. As shown in Figure 5, the integrator circuit uses the op-amp shown in Figure 6 (the block diagram of the 1.2 μm technology operational amplifier in Figure 4). The op-amp is designed according to the specifications described in Table 3.

Figure 6 is, in detail, the block diagram of the 1.2 μm technology operational amplifier in Figure 4 that shows the operational amplifier with CMOS transistors, formed by the stages of a differential amplifier (M1, M2, M4, and M6), gain stage (M7, M91, M101, and M8) and the output stage (buffer) (M9 and M10). Compensation network M13 and capacitor Cc, where M13 is made up of a polarization network by the transistors M14, M15, and M16. The dimensions of the main semiconductor N-channel and P-channel transistors are listed in Table 4. The characteristic values of the integrator are described in Table 5 [18,19].

**Figure 7:**
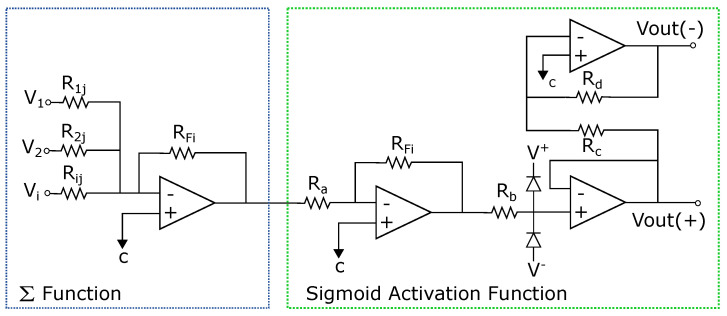
Circuit for one neuron.

The complete circuit of the analog neural network is shown in Figure 3, where the inputs $X_{i}$, the weights (defined by the resistors $R_{ij}=w_{ij}$), and the activation functions (Σ and *F*) are represented. The basic circuits that correspond to the analog neural network neurons are the inverting amplifiers and the activation function. The inverting amplifier circuit is used as a summation block. Hence, when many input voltages are connected to the inverting input terminal, the resulting output is the sum of all the input voltages applied, although inverted; this output, combined with the feedback resistor, generates the multiplication by a weight. The circuit for implementing a neuron is shown in Figure 7, where the function Σ computes multiplications and the activation function is a Sigmoid [19].

As shown in Figure 3, the architecture has 9 neurons (configuration 5-3-1), and each neuron has 4 op-amps due to the adder and the sigmoid activation function (see Table 6). The total power consumption is 46.08 mW, because there are 36 op-amps for the entire circuit, and each of them consumes 1.28 mW [18,20,21].

## 5. Application to an Implemented Analog ANN for Pattern Detection

The ANN-based architecture of this work (see Figure 3) is proposed for pattern recognition as an application example, where the recognition task consists of detecting a value of +1 or −1 for Hamming code correction. One of the advantages of the proposed circuit is that it can be integrated as a module in some communication systems in application-specific integrated circuits (ASIC).

The electronic simulation of the backpropagation neural network is implemented on the basic circuits designed for a typical neuron (the weighted sum function and the activation function). This simulation is carried out using the Pspice program [18], which allows changing the device parameters as well as the stimuli, in such a way that different operating conditions of the circuit are covered.

Once modeled the behavior of the cells that make up the backpropagation, the complete circuit simulation to study the network performance as a whole can continue. This is done by connecting the synapses and the activation functions according to the configuration established in Figure 3. The input vector is bipolar (see Table 7).

**Figure 8:**
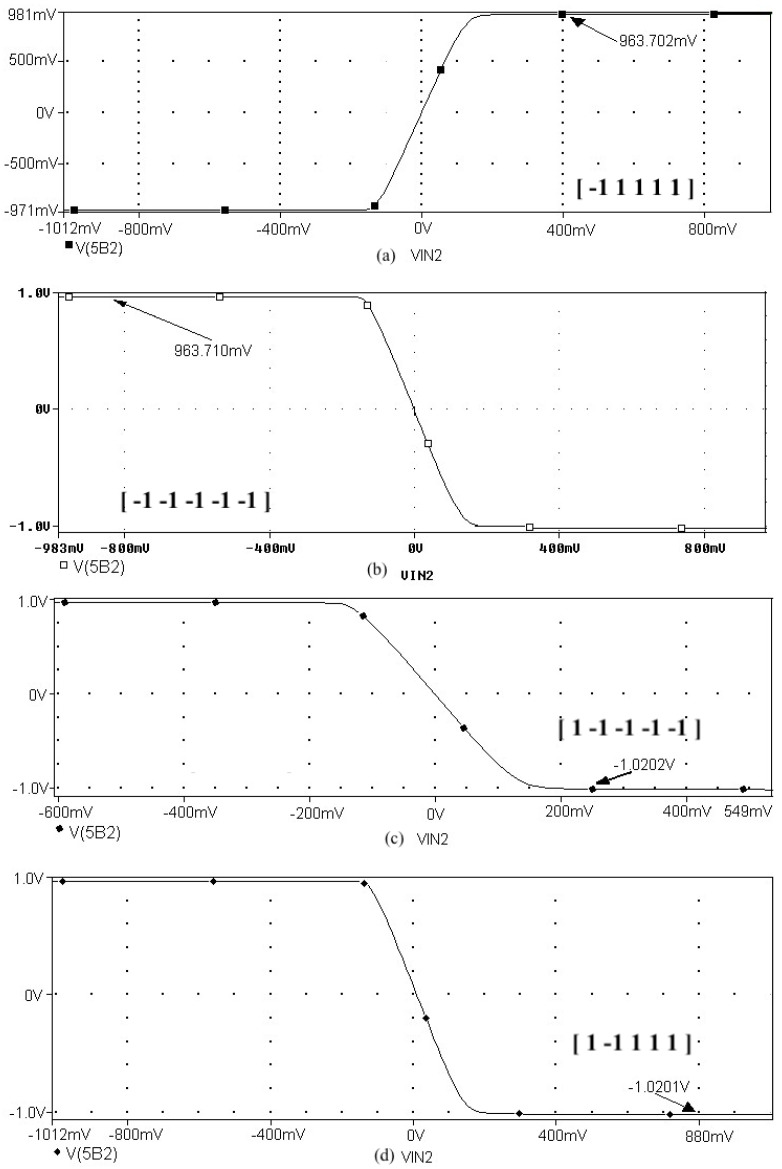
Response of the neural network to different stimulus. The stimulus means external inputs to the network. (**a**) P = [−1 1 1 1 1]; T = [1], (**b**) P = [−1 −1 −1 −1 −1]; T = [1], (**c**) P = [1 −1 −1 −1 −1]; T = [−1], and (**d**) P = [1 −1 1 1 1]; T = [−1].

Figure 8 represents the neural network response (designed to classify input patterns with a supervised learning algorithm) to different stimuli, with a sweep of the output signal concerning the input signal. Figure 8a represents a stimulus P = [−1 1 1 1 1] and the corresponding output T = [1]; if we observe from left to right, we have a negative input and a positive posterior one, which is consistent with the input pattern and its positive output: 1. Figure 8b shows the behavior of the architecture with a stimulus P = [−1 −1 −1 −1 −1] and an output T = [1]; if we sweep the input signal from left to right, we can observe that all the input patterns are negative and we have a positive output at 1. For Figure 8c we have an input pattern P = [1 −1 −1 −1 −1] and an output T = [−1], sweeping from left to right the input pattern is negative and then positive; where the first position of the input vector will be the least significant one for the response of the architecture, resulting in a negative output. Finally, for Figure 8d we have an input pattern P = [1 −1 1 1 1] and an output T = [−1]; if we sweep the output signal from left to right, we observe that the negative value of the pattern is not preponderant for the output of the architecture, which in this case is negative.

The proposed methodology is based on the problem analysis, the datasets definition (inputs and outputs) for a supervised method, the evaluation of ANN configurations, the selection of the best configuration, the implementation of the configuration through operational amplifiers, and the simulation of the ANN-based circuit. In this work, a detection circuit is implemented. The outline training is done through the use of Mathematica (i.e., the training, in which weights and biases are estimated, is a software-based step carried out prior to the execution of the simulation of the ANN-based circuit), and the ANN configuration is carried out using op-amps.

In [22,23], the authors presented a circuit-level implementation of the backpropagation learning algorithm, exploring the gradient descent method in the design of analog circuits. They used an ANN in memristive crossbar arrays, simulating in SPICE on TSMC’s 180 nm CMOS technology, reporting output voltages and timing behaviors.

In our case, we presente an analog system simulation based on the solution to optimization problems with objective functions of one and two variables using the gradient descent method (ascending slope). We showed the ANN configuration behavior (learning algorithm), where its input and process signals are analog. Our circuit-level implementation was made with a 5-3-1 configuration using the backpropagation training algorithm, reporting the simulation-level architecture results in PSpice level 9.1 on 1.2 μm technology with a lambda of 0.5 μm, with widths and lengths of the X-channel defined by the technology.

Although the learning of the neural network was developed offline, and we consider the optimization results obtained in the mathematical part of the article were satisfactory, we emphasize that its essential purpose was to illustrate the applicability to optimize the learning algorithm of an ANN. We also simulated the optimized ANN model through the design of a circuit with CMOS transistors.

## 6. Comparison between Our Proposal and Related Works

Hardware design for neural architectures using base neurons built with CMOS transistors is a fundamental part of our design in comparison with the current cited works. Although we cannot compete in processing speed, we have the advantage of implementing ANN circuits with standard cells without modifying circuits for implementation. We chose several aspects to carry out a comparison among similar works [21,22,23] (see Table 8) considering: (1) the chosen material or device type, (2) the kind of device on which the synapsis is based, (3) the amount of power required, and (4) the learning algorithm used.

The synaptic weights of the proposed method, presented in Table 3, were obtained offline by the Backpropagation learning method for the analog implementations and by a Spiking Neural Network (SNN) for the field-programmable gate array (FPGA) implementation. In our proposal, the synaptic weights were implemented with resistors and op-amp circuits to form the base cell. In reference [21] the synapses were implemented with an array of resistances and op-amps. In contrast, in reference [22] the synapses were implemented by means of cells formed in the Memristive Crossbar. Finally, in the FPGA [23] they were implemented through modules that perform fixed arithmetic calculations using the hardware resources of the family.

A comparison between the proposed work and the implementation using FPGAs is somehow unfair because of the density of programmable logic of the latter, considering energy consumption, processing speed, and clock cycles, among other factors.

Power consumption is directly proportional to the density of the devices used to design the architectures at a hardware level. In the case of our proposal, power consumption is due just to op-amps and resistances. In the case of [22], power consumption is subject to the cells of the transistors, where the effect of signal propagation delay is not negligible. Finally, in the case of the FPGA implementation [23], power consumption was because of the density of programmable logic gates for the whole system; where we should consider static and dynamic powers. For instance, there is a device with unused hardware resources though consuming power since hardly an architecture will consume 100% of the FPGA resources, generating a non-optimal consumption of resources.

The architecture of [23] and our proposal are very different, in our case the architecture presents 9 neurons implemented in parallel and in the case of [23], the authors reported an architecture that calculated the 2842 neurons by using an iterative process (processor-type architecture), where a single neuron was implemented, and this implementation was able to compute the result of all neurons due to the design of the proposal. In this way, if the architecture of [23] implemented 9 neurons, as the same basic architecture requires, it would be consuming similar energy as they reported, since it was a processor architecture that was operating. Implementing 2842 neurons in the FPGA with floating numbers and their processing modules for addition, subtraction, etc., would require greater power consumption and hardware resources, which can be limited by the amount of FPGA resources.

We cannot compare the work in [22] and ours directly, even though both presented simulations carried out in Matlab and Pspice, respectively. For both cases, the technology and implementation were different. However, it is reasonable to think that the better the technology, such as memristors, the lower the energy consumption. In various works, such as in [24], it was said that the energy consumption of [22] was high, and they seeked to lower it.

The work in [22] was developed in 180 nm TSMC’s CMOS technology. The implementation and simulation of the circuit were carried out with memristors and in a crossbar configuration. The authors of [22] implemented a neural network with three neurons in the input layer, two neurons in the output layer, and five neurons in the hidden layer. In contrast, we proposed an ANN made with CMOS with 1.2 μm technology with five neurons in the input layer, one neuron in the output layer, and three neurons in the hidden layer.

Power dissipation was lower in our case because in the CMOS design, being this a mature technology, there are rules that must be satisfied [25,26,27]. On the other hand, in the case of memristors, their implementation in the commercial area is not available. Hence, the silicon organization of their components is still being built or defined. In the case of speed and performance, it is not competitive compared to DRAM, which is used by CMOS technology.

Other factors can affect the behavior of the circuits, but since they are different technologies, their usefulness in the comparison is null, so they are not part of the analysis of this work, such as frequency effects, transistor channel dimensions (W/L), channel resistance, channel conductivity, voltage threshold, response speed, scaling of CMOS devices, reduced geometry effects, channel length, and CMOS channel width.

## 7. Conclusions

In this work, an MLP architecture design at the hardware level was presented, using base neurons at the circuit level as its building blocks. Neuron weights and bias were optimized off-line using n-dimensional objective functions through the gradient descent method. The hardware architecture of the base neuron was designed on a CMOS operational amplifier in 1.2-micron technology. The total power consumption was 46 mW. The 5-3-1 ANN architecture was designed with nine neurons, using 36 op-amps, demonstrating its electronic performance in the practical case shown in Section 5.

The application developed with the implementation of the base neuron circuits showed optimal results in detecting the pattern. Starting with the supervised learning algorithm and based on the gradient descent method to obtain the synaptic weights and bias for the neural architecture, the responses shown in Figure 8 and Table 7 were appropriate to the electrical behavior. The MLP architecture presented an efficiency of 99 %. We might suggest other applications, but in the first instance, we determined that solving this problem was an adequate choice to show the efficiency of the MLP system. Other applications could be proposed, but that remains as future work; for example, we are currently working on image recognition to classify agricultural products and on data processing of hypertensive and diabetic patients. As part of the future work, we have the design and implementation of neural network circuits based on the optimization of objective functions. Besides, we can use other tools, such as Verilog-AMS or VHDL-AMS, as alternatives to simulate the results presented in this work. Another future work is trying other meta-heuristic optimization methods to obtain better ANN training results. We consider that the application of the proposed method was optimal but not without leaving a possibility of applying another meta-heuristic method in the short term.

The advantage of our development with respect to other works cited in Table 8, can be observed, mainly, in the following improvements: power dissipation, number of transistors, and probably, the dimension in area of the integrated circuit. It is likely that one of the disadvantages of our implementation is the speed of response, but that is compensated by the fact that this is a design containing just what is necessary for the application, without an excessive amount of resources.

## Figures and Tables

**Table 1:** Part One: Objective function.

Type of Function	Objective Function	Result
Minimizing the one-dimensional objective function: $E\left(x\right)=2*sin^{2}\left(x\right)+0.1x^{2},x\in R$	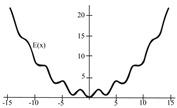	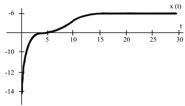 initial conditions x(0)=−15 and $x^{{}^{\prime }}\left(0\right)=10$
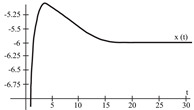 initial conditions x(0)=−12 and $x^{{}^{\prime }}\left(0\right)=10$	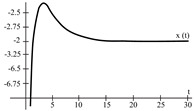 initial conditions x(0)=−9 and $x^{{}^{\prime }}\left(0\right)=10$	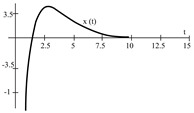 initial conditions x(0)=−6 and $x^{{}^{\prime }}\left(0\right)=10$
Minimizing the n-dimensional objective function: E(x) = $sin\left(x_{1}\right)*sin\left(x_{2}\right),x\in R^{2}$	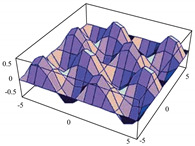	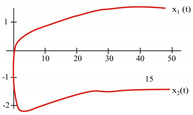

**Table 2:** Part Two: Initial Conditions of the Objective functions.

Type of Function	Initial Conditions of Objective Function
Minimizing the one-dimensional objective function	where $\mu_{0}$ is a scaling factor. The coefficients δ, γ, and T(=1) are constant. For $\delta_{0}=0.1$, and $\gamma_{0}=0.15$, the initial conditions (starting points) are: x(0)= −15, −12, −9, −6, and $x^{\prime }\left(0\right)=$ 10.
Minimizing the n-dimensional objective function	with initial conditions $x_{1}\left(0\right)=-1,\;x_{1}^{{}^{\prime }}\left(0\right)=2,\;x_{2}\left(0\right)=-1,\;x_{2}^{{}^{\prime }}\left(0\right)=-2$.

**Table 3:** Parameters of CMOS op-amp.

Parameter	Value
Open-loop voltage gain	60 dB
Phase margin ($\phi_{o}$)	81${}^{\circ }$
Unity gain bandwidth (GB)	2.30 MHz
Slew rate (SR)	21.06 V/μs
CMRR	47 dB
Offset voltage (Vos)	284 μV
Compensation capacitance (CC)	8.75 pF
Capacitive load (CL)	20 pF
Power supply (VDD, VSS)	+/−2.5 V
Power consumption	1.28 mW
Common mode input voltage range	−608.696 mV to 1.98 V

**Table 4:** Widths and lengths for N-channel and P-channel MOS transistors.

	2 μm Technology		1.2 μm Technology	
	N-Channel MOS		P-Channel MOS	
**Transistor**	**Width**	**Length**	**Width**	**Length**
	Differential Stage			
M1	15 μm	5 μm	9 μm	3 μm
M2	15 μm	5 μm	9 μm	3 μm
M3	70 μm	5 μm	42 μm	3 μm
M4	70 μm	5 μm	42 μm	3 μm
M6	30 μm	5 μm	18 μm	3 μm
M16	15 μm	5 μm	9 μm	3 μm
M14	70 μm	5 μm	42 μm	3 μm
M15	70 μm	5 μm	42 μm	3 μm
	Cascode Stage			
M13	70 μm	5 μm	42 μm	3 μm
M7	70 μm	5 μm	42 μm	3 μm
M91	15 μm	2 μm	9 μm	1.2 μm
M101	70 μm	2 μm	42 μm	1.2 μm
M8	15 μm	5 μm	9 μm	3 μm
	Output Stage			
M9	150 μm	2 μm	90 μm	1.2 μm
M10	700 μm	2 μm	420 μm	1.2 μm

**Table 5:** Characteristic Values of the CMOS integrator circuit.

Characteristic	Values
Slew rate	21.06 V/μs
Cutoff frequency	2.2570 MHz
Gain	60 dB
Phase Margin	83.782${}^{\circ }$
A1 gain	33.33
A2 gain	55.55

**Table 6:** Number of op-amps and total power consumption.

Description	Quantity
No. of op amps per adder	1
No. of op amps per activation function	3
No. of op amps per neuron	4
No. of neurons for the 5-3-1 architecture	9
No. of op amps for the 5-3-1 ANN circuit	36
Total power consumption	46.08 mW

**Table 7:** Bipolar input vector.

Input Vector (P)					Target (T)
−1	−1	−1	−1	−1	1
−1	−1	−1	−1	1	1
−1	−1	−1	1	−1	1
−1	−1	−1	1	1	1
−1	−1	1	−1	−1	1
−1	−1	1	−1	1	1
−1	−1	1	1	−1	1
−1	−1	1	1	1	1
−1	1	−1	−1	−1	1
−1	1	−1	−1	1	1
−1	1	−1	1	−1	1
−1	1	−1	1	1	1
−1	1	1	−1	−1	1
−1	1	1	−1	1	1
−1	1	1	1	−1	1
−1	1	1	1	1	1
1	−1	−1	−1	−1	−1
1	−1	−1	−1	1	−1
1	−1	−1	1	−1	−1
1	−1	−1	1	1	−1
1	−1	1	−1	−1	−1
1	−1	1	−1	1	−1
1	−1	1	1	−1	−1
1	−1	1	1	1	−1
1	1	−1	−1	−1	−1
1	1	−1	−1	1	−1
1	1	−1	1	−1	−1
1	1	−1	1	1	−1
1	1	1	−1	−1	−1
1	1	1	−1	1	−1
1	1	1	1	−1	−1
1	1	1	1	1	−1

**Table 8:** Comparison between our proposal and related works.

Work	Device Quirks	Synapsis Based on	Power Consumption (mW)	Learning Algorithm
Our Proposal	CMOS	op-amps	46.08	BP
Zhang et al. [21]	Memristor	Memristor array + op-amps	-	BP
Krestinskaya et al. [22]	Memristive Crossbar	CMOS	115.65	BP
J. Han, et al. [23]	FPGA	LUT, FF, BRAM	477	SNNs

## Data Availability

Not applicable.
